# Development of a Detection System for *ESR1* Mutations in Circulating Tumour DNA Using PNA-LNA-Mediated PCR Clamping

**DOI:** 10.3390/diagnostics13122040

**Published:** 2023-06-12

**Authors:** Yuki Kojima, Emi Noguchi, Tomomi Yoshino, Shigehiro Yagishita, Shu Yazaki, Hitomi S. Okuma, Tadaaki Nishikawa, Maki Tanioka, Kazuki Sudo, Tatsunori Shimoi, Ayaka Kazama, Hiroshi Terasaki, Sachiro Asano, Yasuhiro Fujiwara, Akinobu Hamada, Kenji Tamura, Kan Yonemori

**Affiliations:** 1 Department of Medical Oncology, National Cancer Center Hospital, Tsukiji 5-1-1, Chuo-ku, Tokyo 104-0045, Japan; yuukojim@ncc.go.jp (Y.K.);; 2 Division of Molecular Pharmacology, National Cancer Center Research Institute, Tsukiji 5-1-1, Chuo-ku, Tokyo 104-0045, Japan; 3 Department of Pharmacology and Therapeutics, National Cancer Center Research Institute, Tsukiji 5-1-1, Chuo-ku, Tokyo 104-0045, Japan; 4 Molecular Genetic Research Department, LSI Medience Corporation, Shimura 3-30-1, Itabashi-ku, Tokyo 174-8555, Japan; 5 Life Technologies Japan Ltd., Thermo Fisher Scientific, Shibaura 4-2-8, Minato-ku, Tokyo 108-0023, Japan

**Keywords:** *ESR1* mutations, breast cancer, peptide nucleic acid and locked nucleic acid polymerase chain reaction, next-generation sequencing, circulating tumour DNA

## Abstract

Although circulating tumour DNA (ctDNA)-based next-generation sequencing (NGS) is a less invasive method for assessing *ESR1* mutations that are essential mechanisms of endocrine therapy resistance in patients with oestrogen receptor-positive breast cancer, adequate amounts of DNA are required to assess polyclonal *ESR1* mutations. By combining a peptide nucleic acid and locked nucleic acid polymerase chain reaction (PNA-LNA PCR) clamping assay, we have developed a novel detection system to screen for polyclonal *ESR1* mutations in ctDNA. A validation assay was prospectively performed on clinical samples and compared with the NGS results. The PNA-LNA PCR clamp assay was validated using six and four blood samples in which *ESR1* mutations were detected by NGS and no mutations were detected, respectively. The PNA-LNA assay results were comparable with those of NGS. We prospectively assessed the concordance between the PNA-LNA PCR clamp method and NGS. Using the PNA-LNA PCR clamp method, *ESR1* mutations were detected in 5 out of 18 samples, including those in which mutations were not detected by NGS due to small amounts of ctDNA. The PNA-LNA PCR clamping method is a highly sensitive and minimally invasive assay for polyclonal *ESR1* mutation detection in the ctDNA of patients with breast cancer.

## 1. Introduction

Oestrogen receptor alpha (ERα), encoded by the *ESR1* gene, is a member of the nuclear hormone receptor superfamily and is expressed in approximately 70% of breast cancer cases [1]. Patients with metastatic oestrogen receptor (ER)-positive breast cancer are treated with a variety of endocrine therapies until they eventually develop endocrine resistance or visceral crisis [2]. Although *ESR1* mutations are rare in primary breast cancers, they are frequently reported in patients with recurrent breast cancer who have previously received endocrine therapy [3,4,5]. Furthermore, *ESR1* mutations have emerged as an essential mechanism of resistance to endocrine therapy and as predictive biomarkers for resistance to therapy and potential therapeutic targets.

With the improvements to next-generation sequencing (NGS) technology, genomic analysis of blood samples from cancer patients has been conducted in various studies [6], and the detection of *ESR1* mutations in circulating tumour DNA (ctDNA) in patients with breast cancer has been reported [7,8,9,10,11]. In addition, the presence of *ESR1* mutations has been reported to impact the efficacy of subsequent treatment modalities [7]. The most frequently occurring *ESR1* mutations are in the ligand-binding domain (LBD) of ERα, generally clustering between amino acids 534 and 538. D538G, Y537S, and E380Q are the major hotspot mutations in ERα LBDs, and others detected include L536Q, Y537N, and Y537C. Preclinical studies have shown that these mutations confer resistance to hormone therapy [5,12,13,14,15]. Polyclonal detection of these genomic hotspot mutations after endocrine therapy has been reported [16]. Evaluating *ESR1* mutations in ctDNA using NGS technology can detect rare mutation sites and polyclonal mutations; however, it requires a sufficient amount of nucleic acid and is time-consuming. In contrast, a digital polymerase chain reaction (PCR) can evaluate hotspot mutations with high sensitivity, as shown in several studies [7,8,10,11,16,17,18]. However, a PCR can only detect mutations at specifically defined sites.

A peptide nucleic acid (PNA)-based PCR is a highly sensitive and specific approach for the detection of genetic mutations. This technique enhances the specificity of the PCR reaction by targeting the initial step involved in nonspecific amplification, namely, primer binding to a mismatched target sequence. PNA is a DNA analogue in which the four nucleotides, adenine (A), thymine (T), guanine (G), and cytosine (C), are attached to an N-(2-aminoethyl) glycine backbone rather than to the negatively charged deoxyribose phosphate backbone that is present in DNA [19]. Genomic analysis using the PNA-locked nucleic acid (LNA)-mediated PCR clamping (PNA-LNA PCR clamp) method has been shown to be highly sensitive in detecting gene mutations in *EGFR* and *RAS* [20,21,22,23]. Although the use of ctDNA is a less invasive and less expensive method for assessing *ESR1* mutations, few studies have evaluated its feasibility compared with that of NGS. The amplified product of clamp PCR can also be directly sequenced to evaluate mutations in the defined region; therefore, this feature was considered useful for assessing polyclonal *ESR1* mutations. To detect polyclonal *ESR1* mutations with simplicity, low cost, and low invasiveness, we developed a novel assay for the detection of polyclonal *ESR1* mutations in ctDNA using the PNA-LNA PCR clamp method. In addition, patient blood samples were prospectively collected to evaluate the feasibility of this system compared with that of NGS.

## 2. Materials and Methods

### 2.1. Design for the Detection of ESR1 Mutations

**Figure 1:**
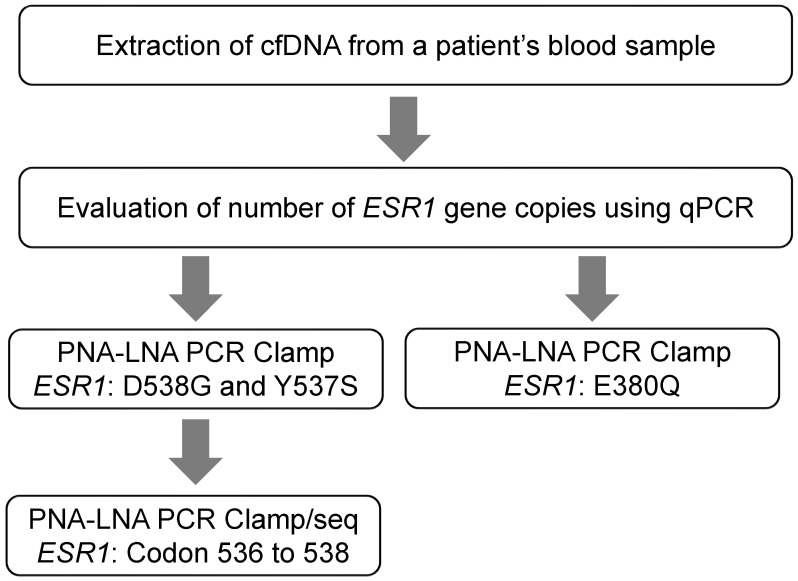
Detection of *ESR1* mutations in ctDNA using a PNA-LNA PCR clamp assay. cfDNA, cell free DNA; LNA, locked nucleic acid; PNA, peptide nucleic acid.

To develop a detection system for *ESR1* mutations using PNA-LNA PCR clamping, we focused on three common hotspot mutations in *ESR1* (E380Q, Y537S, and D538G) that have been reported to be important for acquired resistance to endocrine therapy [7,24,25]. In addition to these three hotspots, other *ESR1* mutations have also been detected at lower frequencies, mainly in the region of codons 536–538 [25]. Therefore, we performed direct sequencing of the 536–538 codon region using amplified products obtained by clamp PCR targeting D538G and Y537S to identify additional mutations in this region (Figure 1).

### 2.2. Plasmids

Wild-type fragments containing the *ESR1* gene were amplified using PCR from normal human genomic DNA (gDNA). The primers used are shown in Table 1. The amplified fragments were cloned into the pGEM^®^ T Easy vector (Promega, Madison, WI, USA), and the plasmid DNA was extracted. Plasmids with *ESR1* mutations were produced by transfection of wild-type plasmid DNA into the template using the PrimeSTAR Mutagenesis Basal Kit (Takara Bio, Kusatsu, Japan).

### 2.3. PNA-LNA PCR Clamp Assay

The number of *ESR1* copies per 2 μL of ctDNA of each sample was calculated based on a standard by real-time PCR. A standard was prepared with 1.0 × 10^2^ to 1.0 × 10^5^ copies from wild-type gDNA. The primers used are shown in Table S1. Real-time PCR was performed using the LightCycler^®^ 480 System II (Roche Life Science, Penzberg, Germany). The thermal cycling profile was 95 °C for 5 min, followed by 50 cycles of 95 °C for 5 s and 62 °C for 30 s. Genomic DNA was purified using a QIAamp DNA Blood Kit (Qiagen, Hilden, Germany).

*ESR1* mutations E380Q, Y537S, and D538G were analysed. PNA-LNA clamp PCR was performed using 4 μL of ctDNA from each patient sample as template DNA. Mutational analyses of Y537S and D538G were performed simultaneously. PNA-LNA clamp PCR was performed using the LightCycler^®^ 480 System II (Roche Life Science, Germany). PCR cycling commenced at 95 °C for 5 min, followed by 50 cycles of 95 °C for 5 s and 62 °C for 30 s.

PNA-LNA clamp PCR products obtained during the mutational analysis of Y537S and D538G were used as template DNAs for amplifying the regions containing codons 536, 537, and 538 using nested PCR. These PCR products were used as template DNA and then sequenced by direct sequencing. The sequencing reagent used was the BigDye Terminator V3.1 Cycle Sequencing kit (Applied Biosystems/Thermo Fisher Scientific, Waltham, MA, USA). Sequencing reaction samples were purified using the BigDye X Terminator Purification Kit (Applied Biosystems/Thermo Fisher Scientific, MA, USA) and then analysed using the PRISM 3730XL (Applied Biosystems/Thermo Fisher Scientific, MA, USA) to detect mutations in codons 536, 537, and 538 of the *ESR1* gene.

The efficiency of PCR clamping was determined by measuring the crossing point (Cp) value. The delta Cp (ΔCp) value was calculated as follows: Cp of sample with PNA—Cp of sample without PNA. ‘Cp of sample without PNA’ was calculated as a conversion from the *ESR1* copy number.

### 2.4. Clinical Samples

**Figure 2:**
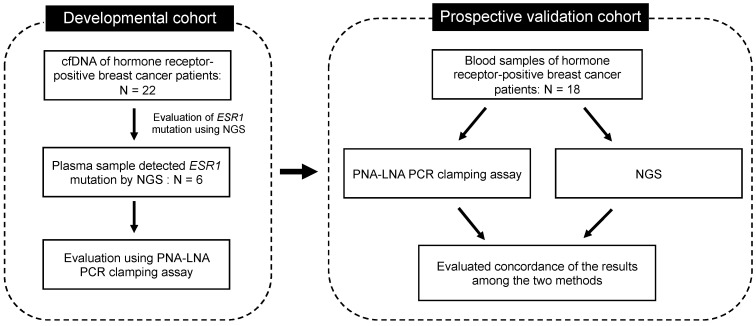
Schematic representation of the developmental and prospective validation cohorts of patients with breast cancer.

To evaluate methods for the detection of *ESR1* gene mutations in blood samples, a prospective study was conducted in patients with recurrent metastatic hormone receptor-positive breast cancer at the National Cancer Center Hospital, Japan, from June 2019 to January 2021. The study included two cohorts: (1) the developmental cohort and (2) the prospective validation cohort (Figure 2). Patient characteristics are shown in Table S1. Patients who consented to the study had 12 mL of blood collected when they were diagnosed with progressive disease (PD) following endocrine therapy or chemotherapy. Ethylene-diaminetetraacetic acid (EDTA) 2K tubes were used as blood collection tubes. Institutional review board and ethics committee approval were obtained from our institutions. This study was conducted in accordance with the Declaration of Helsinki [26], and the study protocol was approved by the Institutional Review Board of the National Cancer Center Hospital, Tokyo, Japan (approval number: 2016-024). All participants provided written informed consent prior to the study-related procedures. This study followed the Strengthening the Reporting of Observational Studies in Epidemiology (STROBE) reporting guidelines (http://www.strobe-statement.org/ (accessed on 29 May 2023)). No statistical methods were used to determine the sample size. Mutations obtained from clinical samples were blinded from the investigators to avoid bias.

### 2.5. Clinical Sample Collection and Cell-Free DNA/TNA Extraction

Plasma samples were collected in EDTA tubes and stored at −80 °C. For the developmental cohort, cell-free DNA (cfDNA) was obtained from collected peripheral blood using the MagMAXTM Cell-Free DNA Isolation kit (Thermo Fisher Scientific, Waltham, MA, USA) according to the manufacturer’s instructions. For the prospective validation cohort, cell-free total nucleic acid (cfTNA) was extracted from blood using the MagMAX™ Cell-Free Total Nucleic Acid Isolation Kit (Thermo Fisher Scientific, MA, USA). The amounts of cfDNA and cfTNA were quantified using the Qubit dsDNA HS Assay Kit (Thermo Fisher Scientific, MA, USA).

### 2.6. Targeted NGS

The method of NGS for evaluating ESR1 mutation in patient samples is shown in the Supplementary Information.

## 3. Results

### 3.1. PNA-LNA PCR Clamp Reaction for the Detection of ESR1 Mutations

**Figure 3:**
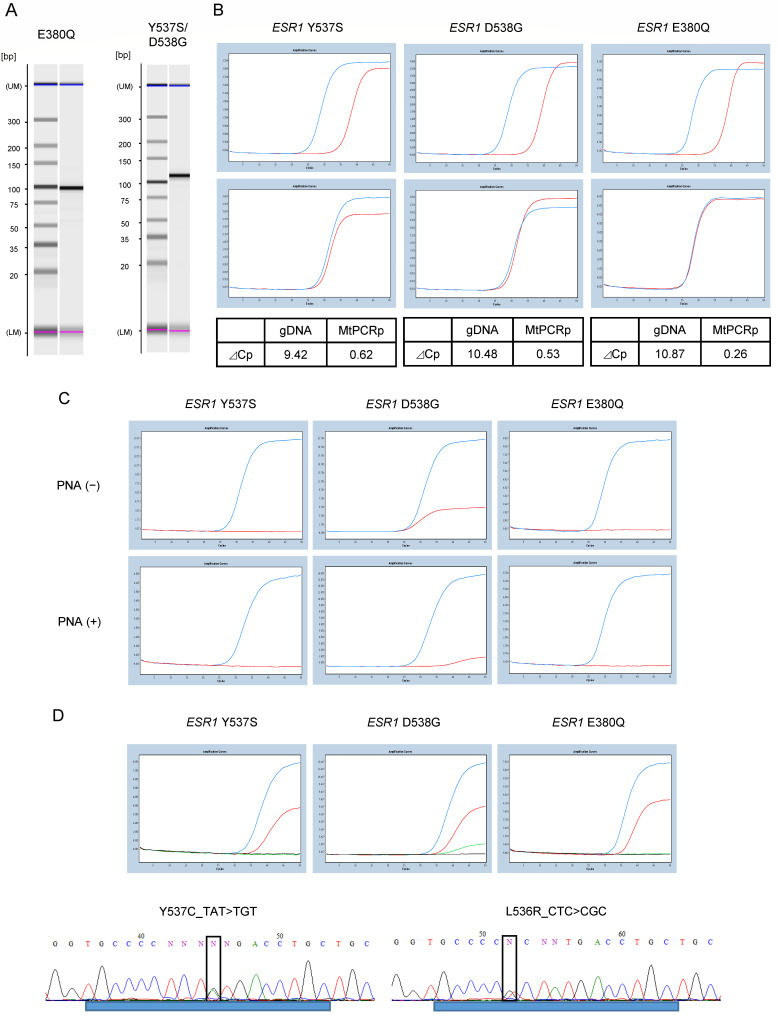
PNA-LNA PCR clamp assay for the detection of *ESR1* mutations. (**A**) Evaluation of primer performance. Amplification of the target product is confirmed by electrophoresis. Specific amplification of the target product is confirmed at an elongation temperature of 62 °C. **Left**: Primer for E380Q, 100 bp. **Right**: Primer for Y537S and D538G, 111 bp. (**B**) Performance evaluation of the PNA clamp assay. Top: wild-type allele (1.0 × 10^4^). Bottom: mutant allele (1.0 × 10^4^). Amplification was suppressed for the wild-type allele although not for the mutant allele. ⊿Cp value is calculated at threshold 0.5. (**C**) Evaluation of the specificity of LNA probes for mutation detection. The upper figure shows the analysis without PNA, and the lower figure shows the analysis with PNA. Blue line: mutant allele; red line: wild-type allele. (**D**) Standard samples of 1% and 0.1% were assayed with the probe to confirm the sensitivity of mutation detection. Analysis of 1.0% (blue line) and 0.1% (red line) of standard samples with mutations. Green line: wild-type *ESR1*; black line: distilled water. The bottom figure shows the sequence results from direct sequencing for 0.1% of the standard sample of Y537C and L536R mutations. ⊿Cp, change in crossing point; LNA, locked nucleic acid; PNA, peptide nucleic acid.

We checked the amplification of the target product using electrophoresis to evaluate the performance of the designed primers. Specific amplification of the target product was confirmed (Figure 3A). We performed PNA clamp assays to confirm that the mutant allele was not amplified, and that the wild-type allele was amplified (Figure 3B). The specificity of the LNA probes for mutation detection was evaluated. Without the addition of PNA, the wild-type gDNA showed a slight signal for D538G, which was suppressed by the addition of PNA (Figure 3C). Finally, the sensitivity of the probes for the detection of *ESR1* gene mutations was evaluated using standard samples (Figure 3D). The probes for all *ESR1* gene mutations showed a detection sensitivity of 0.1%. Standard samples of *ESR1* gene mutations in Y537S and L536R were evaluated by direct sequencing, and mutations were detected with a sensitivity of 0.1%.

### 3.2. Developmental Cohort

We then validated the assay using clinical samples. cfDNA was extracted from the blood samples of patients with hormone receptor-positive breast cancer, and *ESR1* mutations were identified by NGS using the Oncomine pan-cancer cell-free assay. *ESR1* mutations were detected in 6 out of 22 patients. The sites and allele frequencies of the mutations detected in each patient sample are shown in Table S2. Of the six patients with *ESR1* mutations, five had polyclonal mutations. The concordance of detection of *ESR1* mutations between the PNA-LNA PCR clamp method and NGS was evaluated for these six samples with *ESR1* mutations and four samples without *ESR1* mutations. The median Cp value of the samples in which *ESR1* gene mutations were detected was 28.02, compared with a Cp value of 29.70 for samples in which *ESR1* gene mutations were not detected, showing no significant difference (Mann–Whitney U test).

The detection of E380Q, Y537S, and D538G mutations using the PNA-LNA PCR clamp assay was positive in all the samples confirmed by NGS (Table 2, Table 3 and Table 4). We further evaluated whether these mutations were detectable by direct sequencing and found that they were indeed present in all the samples (Figure S1A). The PNA-LNA PCR clamp assay detected a D538G mutation in one of the four samples that did not show any genetic mutations by NGS (Table 4). This specimen was evaluated by direct sequencing and was found to contain a D538G mutation (Figure S1B). Additionally, L536H, Y537N, and Y537C were detected by NGS as hotspot mutations in addition to E380Q, Y537S, and D538G (Table S2). Specimens with L536H mutations also showed Y537S and D538G mutations, and direct sequencing detected Y537S and D538G, although not L536H mutations (Sample #4, Figure S1A). In the two specimens in which Y537N mutations were identified by NGS, one was detected by the PNA-LNA PCR clamp reaction assay, while the other was not (Samples #1 and #3, Figure S1C). The Y537N mutation was not detected in either sample by direct sequencing. Samples with the Y537C mutation were confirmed to have the mutation by direct sequencing (Sample #6, Figure S1D).

Overall, we found that the sensitivity and accuracy of the detection of *ESR1* mutations using the PNA-LNA PCR clamping assay were comparable with those of NGS.

### 3.3. Prospective Validation Cohort

Next, we evaluated whether PNA-LNA PCR clamping could identify *ESR1* mutations as accurately as NGS in patient samples. Eighteen patients with recurrent metastatic hormone receptor-positive breast cancer who developed PD following previous treatment were enrolled, and their blood samples were analysed. The detection of *ESR1* mutations and hotspot regions using PNA-LNA PCR clamping and NGS is shown in Table 5. PNA-LNA PCR clamping detected *ESR1* mutations in 5 out of 18 samples. The recommended amount of cfTNA for the analysis using the Oncomine™ Precision Assay was 1.33 ng/µL (20 µL), and 6 out of the 18 samples met that criterion (Table S3). The two samples (#11 and #15) in which *ESR1* mutations were detected by PNA-LNA PCR clamping and not by NGS had very low quantities of cfDNA (less than 0.5 ng/µL) (Table S4). These results indicate that our method is highly sensitive for detecting *ESR1* mutations in samples where cfDNA levels are too low to be analysed by NGS.

## 4. Discussion

Here, we report a novel method for the detection of *ESR1* mutations in ctDNA using PNA-LNA PCR clamping. Although the PNA-LNA PCR clamp method is highly sensitive, a large number of samples and multiple PCR reactions would be needed to detect a wide range of gene mutations. Because a simple, low-cost, and minimally invasive blood test is desirable for use in clinical practice, we designed an assay method specifically for the detection of high-frequency mutations and mutations associated with therapeutic resistance. The common mutations D538G, Y537S, and E380Q were detected using LNA probes, and additional mutations in codons 536–538 were detected by direct sequencing of the amplified clamp PCR product (Figure 1).

ER-positive breast cancer accounts for approximately 70% of all breast cancers, and endocrine therapies are effective in these patients. Various mechanisms of resistance to endocrine therapies have been reported, including *ESR1* missense mutations [3,4,5]. More than 50 *ESR1* missense mutations have been identified [27]. Although the clinical significance of all hotspot mutations is unknown, *ESR1* mutations in the LBD region are associated with resistance to tamoxifen and fulvestrant [5,13,15,28]. The most common *ESR1* mutations are E380Q, Y537S, and D538G, and these hotspots account for 70% of the *ESR1* mutations [29]. Recently, many studies have reported the detection of *ESR1* mutations using NGS and digital PCR [7,8,9,10,11,16,17,18]. Although NGS is expensive [30,31] and requires high concentrations of cfDNA, it is commonly used to detect polyclonal *ESR1* mutations. We found that our method detected polyclonal *ESR1* mutations with a sensitivity similar to that of NGS and could be performed using only 3 mL of plasma, thus providing a minimally invasive and cost-effective monitoring method. An inexpensive, low-invasive method for detecting ESR1 mutations in breast cancer patients on hormone therapy is very relevant because it could help in identifying patients who may benefit from potential therapeutic options, such as Elacestrant [32], in addition to assessing resistance to hormone therapy.

The PNA-LNA PCR clamp method uses a PNA complementary to the wild-type sequence and a fluorophore-labelled LNA complementary to the mutant sequence to evaluate mutations in the target gene. Because PNA probes form a high-strength double-stranded bond with DNA, a single-base mismatch causes a significant decrease in the melting temperature. In addition, a PNA is less susceptible to displacement by DNA polymerase. Thus, the addition of a PNA sequence complementary to the wild-type sequence inhibits the amplification of the wild-type sequence in the reaction mix. LNA, like PNA, also forms a high-strength double-stranded bond with DNA, and the Tm is greatly reduced by a single-base mismatch. Therefore, the use of a fluorophore-labelled LNA, which is complementary to the mutant sequence, enables the highly sensitive and specific detection of small amounts of mutant sequences in a background of wild-type sequences. While previously reported systems for detecting *EGFR* and *RAS* gene mutations using the PNA-LNA PCR clamp method detect targeted hot spot mutations [20,21,22,23], this analysis can detect mutations in the 536–538 codon region of *ESR1* in addition to the target hot spot mutation. Unlike digital PCR, the PNA-LNA clamp method is qualitative rather than quantitative. Although quantitative evaluation of some mutations, such as *PIK3CA,* has been reported to be significant in predicting treatment response [33], the significance of quantitative evaluation of *ESR1* mutations has not been clarified. However, the *ESR1* mutation is a biomarker that predicts a weaker response to endocrine therapy, and its qualitative detection is considered to be significant.

While our results are promising, it is not entirely clear whether these results can be applied to the detection of all polyclonal *ESR1* mutations in ctDNA. The number of patient samples used in this study was small, and validation of the polyclonal hotspot mutations was limited. In order to evaluate the detection sensitivity for several polyclonal hotspot mutations, it is necessary to validate the system with a larger number of patient specimens. Furthermore, our method cannot detect all potential *ESR1* mutations across all regions of the gene as effectively as NGS.

## 5. Conclusions

In conclusion, our PNA-LNA-mediated PCR clamping assay is a highly sensitive and minimally invasive assay that could be a useful monitoring tool for the detection of *ESR1* mutations in the cfDNA of patients with breast cancer.

## Figures and Tables

**Table 1:** Primer sequences.

Primer	Oligo Sequence, 5′ to >3′
Evaluation of number of ESR1 gene copies	
ESR1-S-F	5′-CAGTAACAAAGGCATGGAGCA-3′
ESR1-S-R	5′-CTAGTGGGCGCATGTAGGC-3′
ESR1-S-Total	5′-Cy5/ACGTTCTTGCACTTCATGCTG/BHQ-3′
Reaction_E380Q	
ESR1 E380Q-AS-F	5′-AGTAGCTTCCCTGGGTGCTC-3′
ESR1 E380Q-AS-R	5′-TGACCCTCCATGATCAGGTCC-3′
ESR1 E380Q-AS-Total	5′-Cy5/CTGATGATTGGTCTCGTCTGGC/BHQ-3′
ESR1 E380Q-AS-LNA	5′-FAM/AGGCACAT + T + G + TAGAAG/BHQ-3′
ESR1 E380Q-AS-PNA	5′-GGCACATTCTAGAAGG-3′
Reaction_Y357S and D538G	
ESR1-S-F	5′-CAGTAACAAAGGCATGGAGCA-3′
ESR1-S-R	5′-CTAGTGGGCGCATGTAGGC-3′
ESR1-S-Total	5′-Cy5/ACGTTCTTGCACTTCATGCTG/BHQ-3′
ESR1 Y357S-S-LNA	5′-FAM/CCCCTC + T+C + TGACCT/BHQ-3′
ESR1 D538G-S-LNA	5′-HEX/CTCTAT + GG + CCTG + CTG/BHQ-3′
ESR1 S-PNA	5′-GCCCCTCTATGACCTGC-3′

**Table 2:** Evaluation of E380Q detection using PNA-LNA PCR clamp reaction.

Sample	Cp Value	Cp Value			E380Q
		PNA+	PNA−	⊿Cp	
#1	N.D.	35.12	26.44	8.68	WT
#2	34.57	33.66	28.46	5.20	Detection
#3	N.D.	33.97	24.37	9.60	WT
#4	N.D.	40.73	29.98	10.75	WT
#5	34.91	33.83	26.93	6.90	Detection
#6	N.D.	39.88	29.78	10.10	WT
Neg_#1	N.D.	37.18	28.44	8.74	WT
Neg_#2	N.D.	39.18	30.24	8.94	WT
Neg_#3	N.D.	39.16	29.98	9.18	WT
Neg_#4	N.D.	36.49	27.67	8.82	WT

Cp, crossing point; N.D., not detected; PNA-LNA PCR, peptide nucleic acid and locked nucleic acid polymerase chain reaction; WT, wild-type. PNA+; analysis with PNA, PNA−; analysis without PNA.

**Table 3:** Evaluation of Y537S detection using PNA-LNA PCR clamp reaction.

Sample	Cp Value	Cp Value			Y537S
		PNA+	PNA−	⊿Cp	
#1	31.92	31.91	25.83	6.08	Detection
#2	N.D.	31.33	27.85	3.48	WT
#3	29.47	29.01	23.76	5.25	Detection
#4	36.66	36.34	29.37	6.97	Detection
#5	N.D.	30.66	26.32	4.34	WT
#6	N.D.	38.28	29.17	9.11	WT
Neg_#1	N.D.	37.71	27.83	9.88	WT
Neg_#2	N.D.	41.64	29.63	12.01	WT
Neg_#3	N.D.	41.93	29.37	12.56	WT
Neg_#4	N.D.	38.32	27.06	11.26	WT

Cp, crossing point; N.D., not detected; PNA-LNA PCR, peptide nucleic acid and locked nucleic acid polymerase chain reaction; WT, wild-type. PNA+; analysis with PNA, PNA−; analysis without PNA.

**Table 4:** Evaluation of D538G detection using PNA-LNA PCR clamp reaction.

Sample	Cp Value	Cp Value			D538G
		PNA+	PNA−	⊿Cp	
#1	32.87	31.91	25.83	6.08	Detection
#2	31.56	31.33	27.85	3.48	Detection
#3	29.84	29.01	23.76	5.25	Detection
#4	36.68	36.34	29.37	6.97	Detection
#5	30.85	30.66	26.32	4.34	Detection
#6	N.D.	38.28	29.17	9.11	WT
Neg_#1	37.92	37.71	27.83	9.88	Detection
Neg_#2	N.D.	41.64	29.63	12.01	WT
Neg_#3	43.26	41.93	29.37	12.56	WT
Neg_#4	39.19	38.32	27.06	11.26	WT

Cp, crossing point; N.D., not detected; PNA-LNA PCR, peptide nucleic acid and locked nucleic acid polymerase chain reaction; WT, wild-type. PNA+; analysis with PNA, PNA−; analysis without PNA.

**Table 5:** Detection of *ESR1* mutations using PNA-LNA PCR clamping compared with NGS.

Sample	PNA-LNA PCR Clamp	NGS
#1	E380Q and D538G	E380Q, L536Q and D538G
#2	WT	WT
#3	WT	WT
#4	WT	WT
#5	WT	WT
#6	WT	WT
#7	E380Q and L536H	E380Q and L536H
#8	WT	WT
#9	WT	WT
#10	WT	NE
#11	Y537S	WT
#12	WT	NE
#13	WT	NE
#14	WT	WT
#15	D538G	WT
#16	WT	WT
#17	Y537S	Y537S
#18	WT	WT

NE, not evaluated; NGS, next-generation sequencing; PNA-LNA PCR, peptide nucleic acid and locked nucleic acid polymerase chain reaction; WT, wild-type.

## Data Availability

Sequence files are available in the NCBI database (PRJNA884163). Additional analysed data files are available in the Supplementary Information of this article.

## Supplementary Materials

The following supporting information can be downloaded at https://www.mdpi.com/article/10.3390/diagnostics13122040/s1, Figure S1: Identification of *ESR1* mutations in clinical samples using PNA-LNA PCR clamp assay; Table S1: Patient characteristics in the developmental and prospective validation cohorts; Table S2: Sites and allele frequencies (%) of *ESR1* mutations detected using next-generation sequencing; Table S3: Gene alterations detected using Oncomine™ Precision Assay; Table S4: Read number and coverage of next-generation sequencing.
