# Supplementary material for: Development of a Detection System for ESR1 Mutations in Circulating Tumour DNA Using PNA-LNA-Mediated PCR Clamping
Source: Diagnostics (Basel). 2023 Jun 12;13(12):2040. doi: 10.3390/diagnostics13122040 (PMC10297184; doi:10.3390/diagnostics13122040)
Supplement: Supplementary file 1 [file diagnostics-13-02040-s001.zip › Supplemental Figure Legends.pdf]

Supplemental Figure Legends

Figure S1. Identification of *ESR1* mutations in clinical samples using PNA-LNA PCR clamp assay (**A**), (**B**), and (**D**) Analysis of *ESR1* mutations in clinical samples using direct sequencing. (**C**) Analytical evaluation by PNA-LNA PCR clamp assay for two samples in which the Y537N mutation was detected by NGS (blue line). Red line: analysis of 1.0% and 0.1% of standard samples with Y537N mutations; green line: wild-type *ESR1*; black line: distilled water. LNA, locked nucleic acid; NGS, next-generation sequencing; PCR, polymerase chain reaction; PNA, peptide nucleic acid.
