# Supplementary material for: Development of a Detection System for ESR1 Mutations in Circulating Tumour DNA Using PNA-LNA-Mediated PCR Clamping
Source: Diagnostics (Basel). 2023 Jun 12;13(12):2040. doi: 10.3390/diagnostics13122040 (PMC10297184; doi:10.3390/diagnostics13122040)
Supplement: Supplementary file 1 [file diagnostics-13-02040-s001.zip › Supplemental_Figure_S1.pptx]

## Slide 1
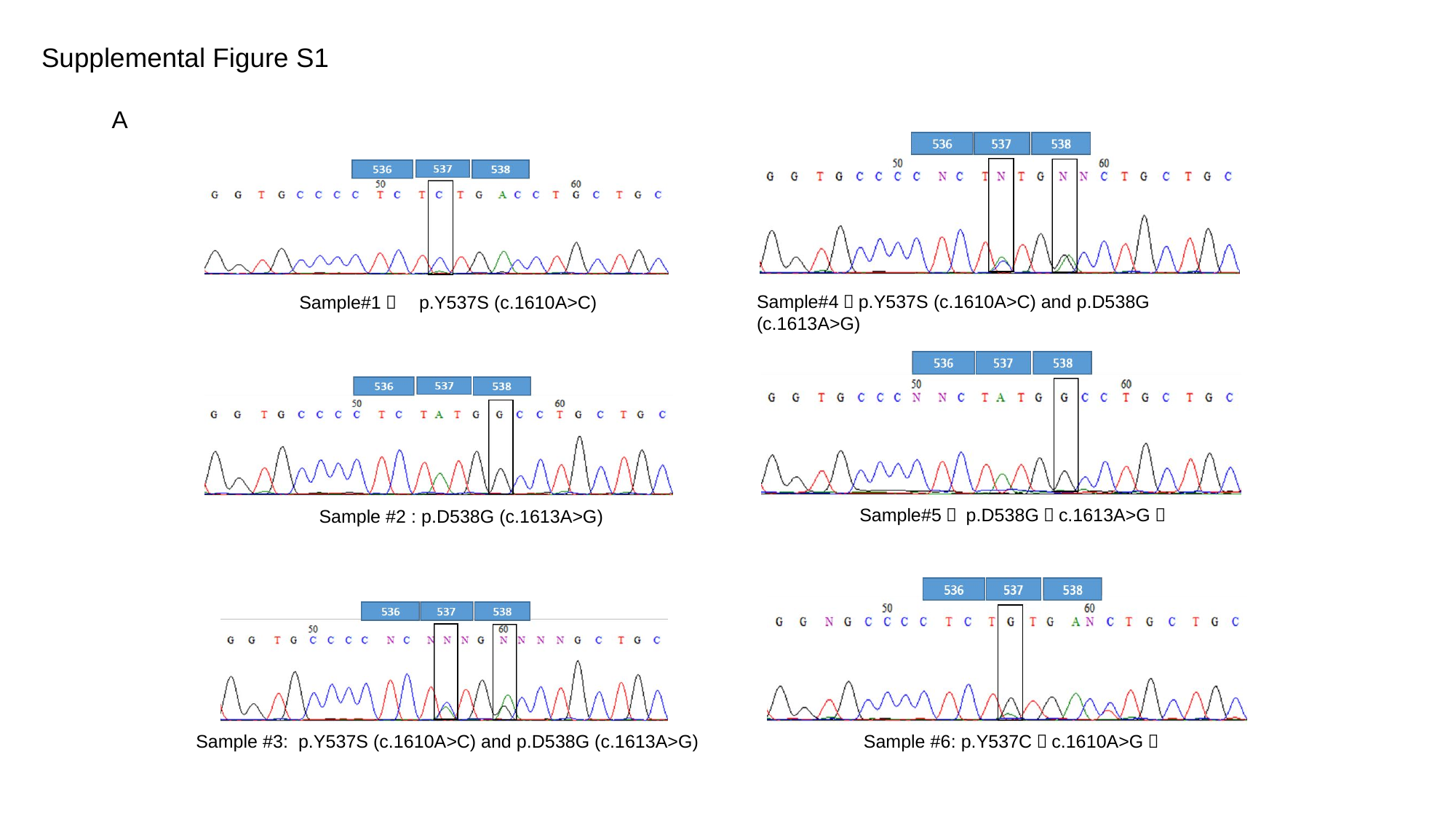

Supplemental Figure S1
A
Sample#4：p.Y537S (c.1610A>C) and p.D538G (c.1613A>G)
Sample#1：　p.Y537S (c.1610A>C)
Sample#5： p.D538G（c.1613A>G）
Sample #2 : p.D538G (c.1613A>G)
Sample #3: p.Y537S (c.1610A>C) and p.D538G (c.1613A>G)
Sample #6: p.Y537C（c.1610A>G）

## Slide 2
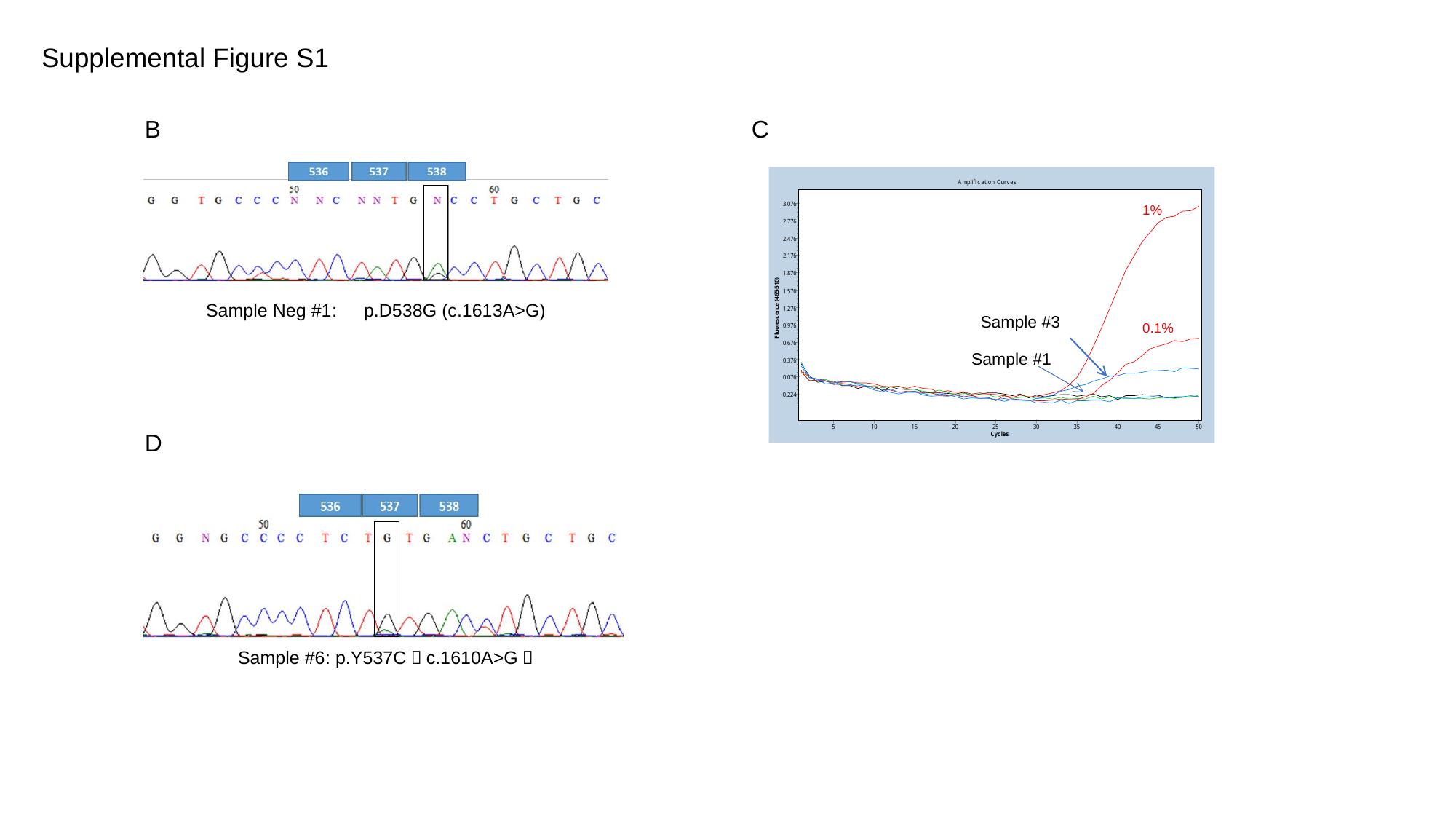

Supplemental Figure S1
B
C
1%
0.1%
Sample #1
Sample #3
Sample Neg #1:　p.D538G (c.1613A>G)
D
Sample #6: p.Y537C（c.1610A>G）
