# Supplementary material for: Development of a Detection System for ESR1 Mutations in Circulating Tumour DNA Using PNA-LNA-Mediated PCR Clamping
Source: Diagnostics (Basel). 2023 Jun 12;13(12):2040. doi: 10.3390/diagnostics13122040 (PMC10297184; doi:10.3390/diagnostics13122040)
Supplement: Supplementary file 1 [file diagnostics-13-02040-s001.zip › Supplemental_Tables_S1-S4.pdf]

Table S1. Patient characteristics in the developmental and prospective validation cohorts.

	Developmental Cohort (N = 22)	Prospective Validation Cohort (N = 18)
Age, years, median (range)	58 (38-71)	66 (49-81)
Gender, female	22 (100%)	18 (100%)
Prior aromatase inhibitors	18 (81.8%)	17 (94.4%)
Prior CDK4/6 inhibitors	15 (68.1%)	12 (66.6%)
Prior chemotherapy	21 (95.5%)	14 (77.8%)

N: number of patients.

Table S2. Sites and allele frequencies (%) of *ESR1* mutations detected using next generation sequencing.

Sample	E380Q	L536H	L536R	Y537N	Y537S	Y537C	D538G
#1	WT	WT	WT	0.1531	2.144	WT	0.4288
#2	1.87	WT	WT	WT	WT	WT	15.36
#3	WT	WT	WT	1.0216	2.5823	WT	2.9512
#4	WT	0.2941	WT	WT	0.4902	WT	1.5686
#5	0.2271	WT	WT	WT	WT	WT	9.3741
#6	WT	WT	WT	WT	WT	0.345	WT

WT: wild-type.

Table S3. Gene alterations detected using Oncomine™ Precision Assay.

Sample	Mutation Variant (Allele Frequency)	Copy Number Variation (/copy)
#1	ESR1 E380Q (57.71%), ESR1 L536Q (0.12%), D538G (19.13%) and KIT D816N (0.10%)	WT
#2	WT	WT
#3	PIK3CA M1043I (1.28%)	WT
#4	TP53 Y234N (0.20%) and TP53 V173M	WT
#5	KRAS Q61H (0.23%), TP53 R175H (0.09%) and EGFR V769M (0.07%)	WT
#6	ERBB3 V104M (0.08%)	WT
#7	ESR1 E380Q (5.14%) and ESR1 L536H (0.22%)	WT
#8	AKT1 E17K (23.78%) and TP53 E285K (47.72%)	FGFR1 (9.27)
#9	PIK3CA P104L (2.24%) and PIK3CA H1047R (1.47%)	WT
#11	WT	WT
#14	PIK3CA K111N (1.25%)	WT
#15	WT	WT
#16	PIK3CA E545K (3.42%)	WT
#17	ESR1 Y537S (0.17%), ERBB2 L755S (7.41%), TP53 R175H (0.17%), PIK3CA H1047R (4.05%)	WT
#18	TP53 M246I (3.59%	PIK3CA (3.06)

WT: wild-type.

Table S4. Read number and coverage of next generation sequencing.

Sample	cfTNA Conc. (ng/μL)	Reads	Average Base Coverage	Median Molecular Coverage per Amplicon
#1	50	12,067,297	43,464	2825
#2	12.9	10,927,842	36,666	2357
#3	0.604	9,190,372	30,353	681
#4	0.868	9,303,902	31,769	1343
#5	10.1	10,077,147	33,329	2851
#6	18.4	9,232,677	27,636	2279
#7	12.7	8,999,667	29,685	1929
#8	0.952	7,258,785	21,012	851
#9	0.776	7,845,123	23,552	902
#10	< 0.20	No analysis due to low concentration		
#11	< 0.50 (0.254)	5,065,195	12,558	301
#12	< 0.20	No analysis due to low concentration		
#13	< 0.20	No analysis due to low concentration		
#14	0.674	7,267,334	22,250	907
#15	< 0.50 (0.250)	4,850,365	11,820	344
#16	0.694	9,036,420	30,750	1167
#17	68.2	9,001,572	29,483	1871
#18	0.736	7,152,730	20,722	845

cfTNA; cell-free total nucleic acid, Conc.; concentration.
